# Epiretinal Membrane and Vitreomacular Traction Associated With Vasoproliferative Tumor in Macular Telangiectasia Type 2: A Case Report

**DOI:** 10.7759/cureus.34612

**Published:** 2023-02-03

**Authors:** Reem Alahmadi, Tariq A AlZahem, Valmore A Semidey

**Affiliations:** 1 Vitreoretinal Division, King Khaled Eye Specialist Hospital, Riyadh, SAU; 2 Ophthalmology Department, College of Medicine, King Saud University Medical City, Riyadh, SAU

**Keywords:** vitreomacular traction, vasoproliferative tumor, mactel, foveal telangiectasia, epiretinal membrane

## Abstract

We report a case of a 62-year-old man who presented with blurred and distorted vision in both eyes. Fundus examination revealed a fibrous band-like membrane emanating from the disc to the foveal center in the right eye, aneurysmal gray parafoveal lesions in both eyes, and an inferotemporal peripheral vascular tumor in the right eye. The presence of an epiretinal membrane with vitreomacular traction in this patient has led to the diagnosis of an incidental peripheral vascular tumor. To our knowledge, there are no reports describing an association between macular telangiectasia type 2 and epiretinal membrane formation with vitreomacular traction due to a vasoproliferative tumor.

## Introduction

In 1982, Gass and Oyakawa first described the clinical entity formerly known as idiopathic juxtafoveal retinal telangiectasia (IJFT), currently known as macular telangiectasia (MacTel) [1]. This is a relatively uncommon group of diseases that causes visual deterioration in the middle-aged population by evolving retinal capillary abnormalities in foveal and parafoveal areas, including incompetence, ectasia, and/or irregular dilations affecting one or both eyes. These changes are usually visualized by fluorescein angiography (FA) exhibiting right-angled vessels and superficial crystalline residues. Gass and Blodi subdivided MacTel into three well-defined groups (types 1, 2, and 3) [2]. Type 1 presents as unilateral retinal telangiectasia in younger patients and has a lesser impact on visual function. Type 2 MacTel is a bilateral juxtafoveal telangiectasia; it is frequently located temporally to the macula and has a symmetric appearance in both eyes. It is typically correlated with a progressively slow deterioration of central vision. In 2006, Yannuzzi et al. suggested a modified classification using modern optical coherence tomography (OCT). Type 1 is termed aneurysmal telangiectasia, which is equivalent to IJFT group I, and type 2 or perifoveal telangiectasia is equivalent to IJFT group II [3]. The literature on epiretinal membranes associated with MacTel is scant.

Vasoproliferative tumors of the retina (VPTR) are vision-threatening, benign, retinal lesions of unidentified origin, mostly affecting patients of ages between 40 and 60 years who are otherwise healthy [4]. These pink to yellow in color lesions on fundoscopic examination are accompanied by intra-retinal hemorrhages, exudates of which are either intraretinal and/or subretinal, and hyperpigmentation of the retinal pigment epithelium. The pathogenesis of these lesions is yet to be clarified. VPTR can be primary or occur secondary to vascular diseases like sickle cell retinopathy and retinopathy of prematurity (ROP), uveitic entities like retinochoroiditis, or other retinal pathologies like retinitis pigmentosa and long-standing retinal detachment [5]. To our knowledge, there are no reports describing an association between MacTel type 2 and epiretinal membrane with vitreomacular traction (VMT) formation due to a vasoproliferative tumor.

## Case presentation

**Figure 1:**
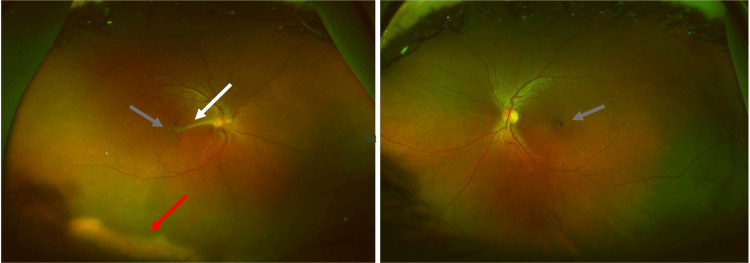
Fundoscopy of the right eye (left image) revealed an epiretinal membrane over the disc attached to the foveal center (white arrow) with concurrent inferior subretinal exudates (red arrow). Bilateral right-angled vessels and grey pigmentary changes can be observed parafoveally (grey arrows).

A 62-year-old diabetic man, not known to have any other systemic illnesses, first presented to our center for distorted vision in both eyes and a gradual decrease in vision in the right eye for over two years, not associated with floaters, flashes, or pain. His ocular history was unremarkable, with no prior reports of trauma or surgery. The best-corrected visual acuity was 20/200 and 20/60 in the right and left eyes, respectively. The slit-lamp examination results were insignificant for both eyes. Fundus examination of the right eye revealed a clear vitreous, healthy optic disc, and a thick fibrous membrane over the disc causing retinal vascular distortion, which extended in a string-like shape and was attached to the fovea, causing VMT. Juxtafoveal vertically oriented, slightly dilated, right-angled venules draining the telangiectatic area were evident biomicroscopically and were associated with gray and temporal parafoveal lesions. Incidentally, inferior subretinal exudates and infratemporal intraretinal hemorrhages, indicating the presence of a VPTR, were observed (Figure 1).

**Figure 2:**
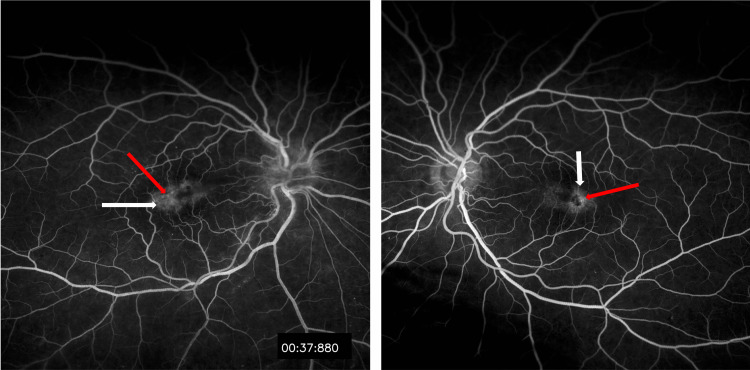
Fluorescein angiography of both eyes showed perifoveal capillary leakage (white arrows) and aneurysmal dilation (red arrows).

**Figure 3:**
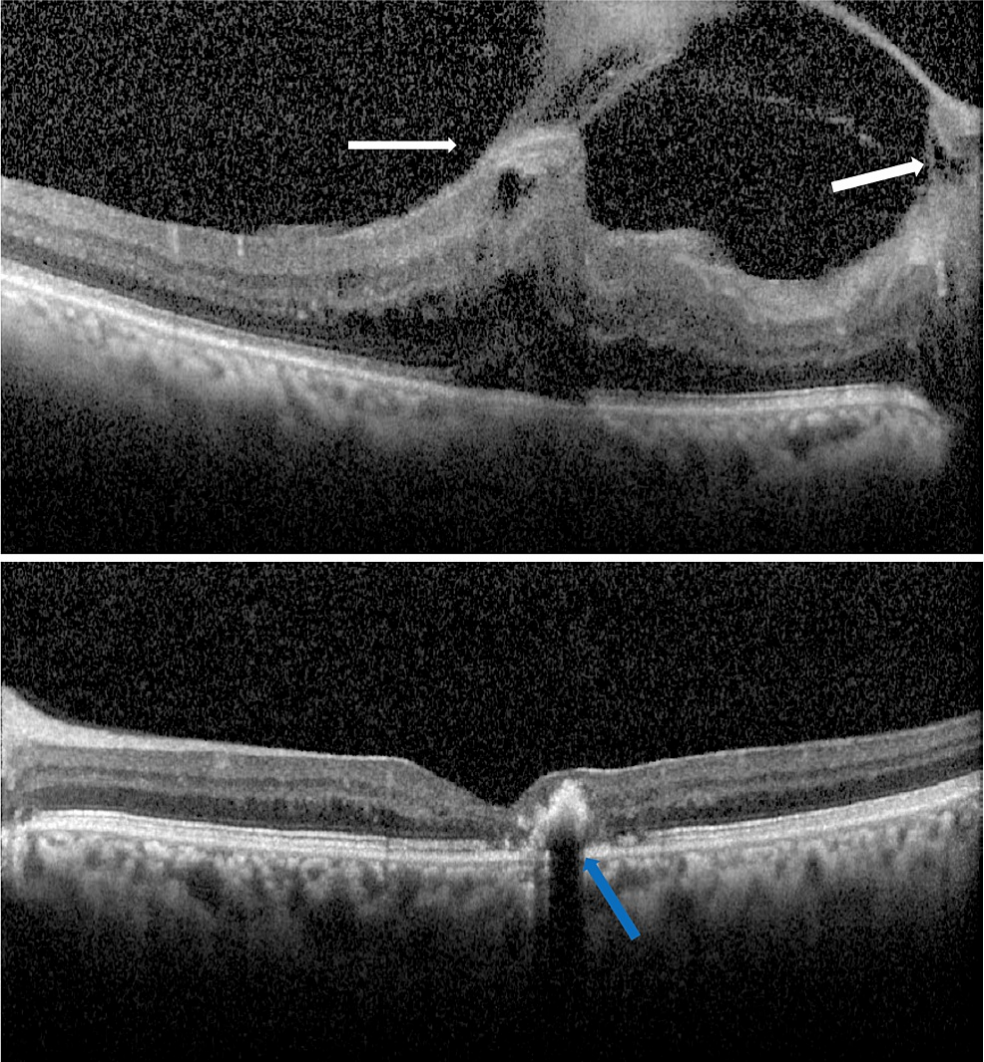
Optical coherence tomography showed vitreomacular traction of the right eye (top image) caused by a gliotic membrane attached to the foveal center and optic nerve head (white arrows). Left eye (bottom image) foveal atrophy and a temporal hyper-reflective lesion corresponding to the pigmentary changes (blue arrow) can be observed.

The left eye was otherwise normal except for the presence of dilated right-angled venules and grey-pigmented parafoveal lesions indicating type II juxtafoveal retinal telangiectasia. FA revealed capillary leakage in both eyes in the perifoveal areas (Figure 2). The fibrous membrane caused VMT on OCT (Figure 3).

Triamcinolone and brilliant blue-assisted pars plana vitrectomy, membrane peeling, and endolaser photocoagulation of the VPTR were performed. Sulfur hexafluoride (SF6) gas was used as an intraocular tamponade at a concentration of 20%. Postoperatively, the patient tolerated the procedure well. The vision improved from 20/200 to 20/100 at one month postoperatively.

**Figure 4:**
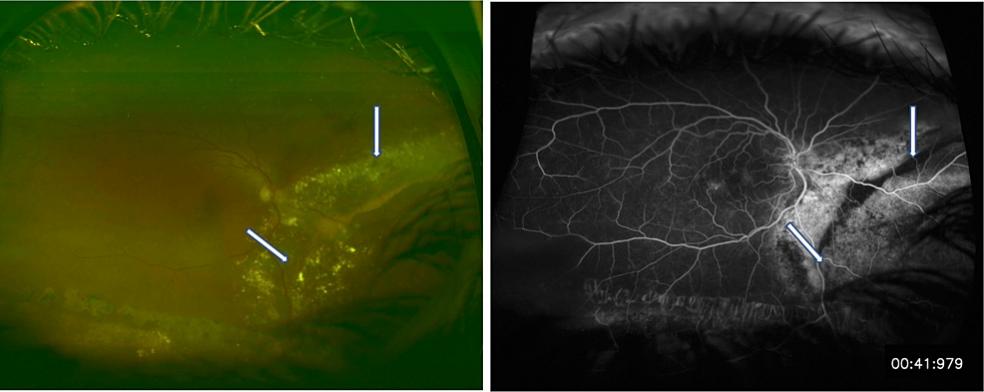
Fundoscopy and fluorescein angiography of the right eye showed progressive subretinal exudates that developed postoperatively (white arrows).

Five months after the surgery, the subretinal exudates increased inferiorly and nasally in the right eye. FA showed extensive inferior and nasal leakage, indicating an active VPTR (Figure 4).

Intravitreal bevacizumab was given, but progressive exudation continued; eventually, triamcinolone acetonide was injected. One month later, partial resolution of the subretinal fluid and exudates was observed. Laser augmentation was performed to manage the VPTR using indirect ophthalmoscopy. Following treatment, the patient’s best-corrected visual acuity stabilized to 20/50 and 20/40 in the right eye and left eye, respectively.

## Discussion

MacTel is thought to be related to capillary malformations leading to nutritional deprivation with subsequent retinal tissue and Müller cell loss, eventually resulting in the abnormal re-modeling of retinal capillaries along with the migration of retinal pigment epithelium (RPE) cells into the retina [3]. Type 2 is the common form of MacTel and differs completely from MacTel type 1. It is acquired and affects middle-aged or older (mean 55 years) individuals with no sex predilection [3]. This bilateral disorder may be asymmetric or manifest unilaterally in its early stages [3]. Gass and Blodi subdivided the natural course of MacTel type 2 into five stages. However, Yannuzzi’s modern OCT-driven classification divides them into two categories: proliferative and non-proliferative perifoveal telangiectasias [3]. Few reports found an association of an epiretinal membrane with MacTel [6,7]. Recently, Ayachit et al. demonstrated an association of epiretinal neovascular membranes with the help of OCT angiography in seven eyes out of 68 patients known to have MacTel. OCT angiography showed a unique vascular proliferation at the level of vitreoretinal interface distinct from the pathognomonic dilated and ectatic capillaries in the superficial and deep vascular plexus that is typical of MacTel [8]. Our patient, unfortunately, had an OCT angiography taken only after the vitrectomy; therefore, this finding could not be observed. The presence of an epiretinal membrane causing VMT has led to the incidental finding of an inferotemporal vasoproliferative tumor in our case. Secondary epiretinal membranes result from a previously existing ocular pathology that could be either vascular or not, central or branch retinal vein occlusion, diabetic retinopathy, retinal breaks with or without detachment, as well as uveitis. Epiretinal membranes are known to be highly associated with vasoproliferative tumors, and 20-31% of VPTR develop macular epiretinal membranes [4,9].

## Conclusions

MacTel presents in a variety of forms, and clinicians and surgeons should be aware of its range of manifestations. Neovascularized epiretinal membranes could provide some insight into the progression of MacTel into advanced stages. The presence of an epiretinal membrane with VMT should alert the physician to thoroughly examine the patient for a primary cause. To our knowledge, this presentation has not been reported in the literature, and further studies are needed to analyze the histopathologic features of these lesions.
